# Silicon Supplementation of Rescuegrass Reduces Herbivory by a Grasshopper

**DOI:** 10.3389/fpls.2019.00671

**Published:** 2019-05-24

**Authors:** Showkat Hamid Mir, Irfan Rashid, Barkat Hussain, Zafar A. Reshi, Rezwana Assad, Irshad A. Sofi

**Affiliations:** ^1^ Department of Botany, University of Kashmir, Srinagar, India; ^2^ Division of Entomology, Sher-e-Kashmir University of Agricultural Sciences and Technology of Kashmir, Srinagar, India

**Keywords:** herbivory, phytolith, grass, mandible wear, silicon

## Abstract

The theory of coevolution suggests that herbivores play an important role in the diversification and composition of plant communities. A prevalent idea holds that grasses and grazing animals participated in an evolutionary “arms race” as grassland ecosystems started spreading across the continents. In this race, besides other things, silicification in the form of phytoliths occurred in the grasses, and the graminivorous herbivores responded through specialized mandibles to feed on plants rich in phytoliths. It is important to understand whether these mandibles equip the herbivores in different environments or the grasses can augment their defense by channelizing their energy in high resource milieu. Here we used rescuegrass (*Bromus catharticus*; Family: Poaceae), an alien species of South America, to understand the mechanism of resistance offered by this species against a local insect herbivore (*Oxya grandis*; Family: Acrididae), graminivorous grasshopper, in different silicon-rich environments. We used different concentrations of silicon and observed the types of phytoliths formed after Si amendments and studied the effect of phytoliths on mandible wear of the grasshopper. Silicon concentrations increased ca. 12 fold in the highest supplementation treatments. The results reveal that higher foliar silica concentration in Si-rich plants did not result in changing the morphology of the phytoliths; still the leaf tissue consumption was lower in higher Si treatments, perhaps due to mandibular wear of the grasshoppers. The study opens a new dimension of investigating the role of Si amendments in reducing herbivory.

## Introduction

The grasses (Poaceae) being the fifth most diverse family of angiosperms (800 genera and more than 11,000 species) have attracted the attention of paleoecologists, particularly in respect of their evolution and diversification (Strömberg, 2005, 2011; Bouchenak-Khelladi et al., 2010; Strömberg et al., 2013; Chen et al., 2015). One prevalent idea is that the grasses and their herbivores diversified by participating in an evolutionary “arms race” during the late Cretaceous – Cenozoic era (Stebbins, 1981). The theory of coevolution proposes that the adaptations between plant species and their herbivores are reciprocal, wherein the plant anti-herbivore traits play a major role in determining the host preference and community structure (Endara et al., 2017). According to this hypothesis, the open-habitat grasses significantly augmented silicon accumulation in the form of phytoliths over time, and to counter tooth wear from grass phytoliths, the mammalian herbivores evolved hypsodont teeth (Stebbins, 1981; McNaughton et al., 1985).

Phytoliths are microscopic amorphous silica bodies that occur as individual cell infillings to wholly silicified tissue sections, which toughen the plant tissues (averting food intake and digestion) and wear the herbivore mouthparts (affecting their normal life) (Strömberg et al., 2016). Although, the actual capacity of grass phytoliths to wear dental tissues and their linkage to hypsodonty evolution has limited experimental evidence (Damuth and Janis, 2011) and has generated much debate more recently (Sanson et al., 2007; Lucas et al., 2013, 2017; Rabenold and Pearson, 2014; Rabenold, 2017), the role of silica-laden abrasive grass diet in the development of mandibles has been suggested in several insect taxa (Chapman, 1964; Dravé and Lauge, 1978; Patterson, 1983, 1984). Elevated mandibular wear due to increased hardness of leaves has been found in various beetles (Raupp, 1985; Wallin, 1988; King et al., 1998), bees (Michener and Wille, 1961; Kokko et al., 1993; Schaber et al., 1993), caterpillars (Korth et al., 2006), a locust (Zouhourian-Saghiri et al., 1983), a weevil (Barnes and Giliomee, 1992), and a bug (Roitberg et al., 2005); and the wear in lepidopteran larvae fed on rice cultivars has been specifically ascribed to differences in silica contents (Djamin and Pathak, 1967; Dravé and Lauge, 1978; Ramachandran and Khan, 1991). However, these experiments used the model interactions in which the plants commonly coevolved with their insect herbivore. Here we tested the model system in which there was a lack of shared evolutionary history between plants and herbivores, creating a novel interaction, wherein the insects were less equipped to face the evolutionary arms race. We selected *Bromus catharticus* Vahl., an alien grass species, and a native herbivore *Oxya grandis* Willemse for the study.

*B. catharticus* is a densely tufted, robust annual or short-lived perennial, native to South America, recently reported as an alien introduction to the flora of Kashmir Himalaya, with the potential to spread along the length and breadth of this biodiversity hotspot (Muzafar et al., 2016). Its large openly branched seed-heads have a nodding appearance, and the glumes generally do not have any awns, while the florets usually have short awns, which make it distinct from other members of genus *Bromus*. On the other hand, *O. grandis* is considered to be a grass-feeding generalist herbivore, hopping in and around the rice fields and grasslands of the study area (Reshi, 2007). *O. grandis* is a large species (over 30 mm) with fully developed tegmina which are extended beyond apices of hind femora. The supra-anal plate is flat, with the apical part lobe extended posteriorly. We hypothesized that:

1. Amount of silica accumulation by plants depends on the presence of available silicon in the soil.
2. Silica polymerizes in the form of phytoliths with different shapes within the cells of *B. catharticus*.
3. Increased exposure of insect herbivores to silica-rich plants will lead to increase in deleterious effects by affecting the insect mandible wear.

Hence, we performed the experiment at various Si concentrations, and expected silica-polymerized phytoliths within *B. catharticus* in high Si environments will affect the mandibles of *O. grandis*.

## Methodology

### Plant Growth Conditions

*B. catharticus* seeds obtained from Integrated Grass Fodder Research Institute (IGFRI) Srinagar, India, were grown in seed trays for 2 weeks containing inert growth media (perlite). Then, the seedlings were transplanted into earthen pots (12 cm diameter × 18 cm height) filled with peat. Peat with its low silicon content is preferred as growth substrate as it provides a better control for the treatments in such environments where additional silicon can be supplemented (Nanayakkara et al., 2008). Four seedlings were planted in a pot at an equal distance from the edge of the pot. The experiment was conducted in a completely randomized design (CRD) under greenhouse conditions (15–25°C, 16:8 light:dark) for a period of 7 months till harvest.

After every 4 days, the plants from the high (T4), moderate (T3), and low (T2) soil-silicon treatments received 50 ml of 2,000, 1,000, and 500 mg/L sodium silicate (Na_2_SiO_3_·9H_2_O) aqueous solution respectively. Plants from the control treatment received the same amount of tap water. After the third week, plants in all treatments were supplemented with 100 ml of half-strength Hoagland’s nutrient solution, which was continuously added till the end of the fifth week. After the sixth week, 100 ml of full-strength Hoagland’s solution was given to all the plants. Throughout the experiment, all plants received tap water as per requirement.

### Si Analysis

The optical emission spectroscopy of atoms excited by inductively coupled plasma (ICP-OES), which is currently one of the most efficient methods for the quantitative determination of elements in materials, was used to detect the Si concentration. The method is characterized by low detection limits and a high selectivity combined with good reproducibility and accuracy. In the present study, the ICP-OES data were recorded by a SPECTRO ARCOS EOP (Germany), spectrometer. Plants were harvested at maturity and the oldest leaves were used for Si estimation. Leaf samples were ground in an electric grinder, and put in a crucible within an incinerator at 800°C for ashing. The ash was then dissolved with aqua-regia and diluted to a known volume using distilled water. A known quantity of the solution was taken in a beaker, and HNO_3_ was added and heated. When it started boiling, perchloric acid (HClO_4_) was added to it dropwise and heated till all the organic matter was destroyed. The solution was then diluted to a known volume, using distilled water. This analytical solution was directly injected into the hot argon ICP plasma (6,000–8,000 K). The spectral line at 251.611 nm which is characteristic for Si was used for the determination of the element concentration. A commercial standard solution of Si was used for calibration of the different concentrations of Si in order to generate a standard curve.

### Phytolith Types

The harvested plants from different treatments were washed with distilled water and chopped into small pieces and then placed in labeled centrifuge tubes (50 ml). The tubes were rinsed with double distilled water before oven drying the material to constant weight. The weighed samples (2 g) were transferred to porcelain crucibles. The plant material was burned for 4–6 h in a muffle furnace at 550°C. The ensuing ash was mixed with 10 ml of hydrogen peroxide (30%) and kept at 80°C for 1 h in a water bath. The mixture was washed twice with double distilled water (DW). The pellet was treated with 10 ml of 10% hydrochloric acid (1 M) and incubated at 80°C for 1 h. The mixture was washed with DW and centrifuged at 3,500 rpm for 15 min. The supernatant was poured off and the pellet was rinsed with DW, till the pellet became clear. Small amount of the dried ash was mixed with 10 ml of Gentian Violet and a drop of this mixture was put on a glass slide which was subsequently covered by a cover slip. Extra stain was drained off with a filter paper and the slide was heated gently. Phytolith morphotypes were observed under a compound microscope (Leica DM300, Wetzlar GMBH) fitted with a digital camera (DFC 320), and photographed at a uniform magnification (40×). Classification of the morphotypes extracted through this dry ashing method was done as per ICPN 1.0 (Madella et al., 2005).

### SEM Analyses of Grasshopper Mandibles

The laboratory colony of grasshoppers (*O. grandis*) was maintained on an artificial diet in the insect-rearing conditions (25–27°C, 14:10 light: dark, 50–60% RH) of the Division of Entomology at SKUAST, Kashmir. These laboratory-reared grasshoppers (third instar stage) were individually caged (*n* = 10) in 1-L sandwich boxes and starved for 24 h, much longer than the clearance time of grasshoppers, so that all food eaten before would have passed through their guts.

Pots from different Si treatments (50 replicates × 4 treatments) were enclosed in muslin cloth bags. Half of the pots (*N* = 100) were exposed to herbivory (infested) by two individuals per pot of third instar nymphs of *O. grandis* for 20 days. After 20 days of infestation under different Si regimes, the adult grasshoppers were collected and treated with 70% ethanol. The preserved mandibles were detached from the mouth part and were cleaned in an ultrasonic shaker. Different concentrations of ethanol, i.e., 80, 90, and 95% were used for dehydration of mandible specimens. In order to make the samples conductive, they were mounted on sample stubs, and then coated with gold for 5 min using a gold sputter coater. Following coating, the samples were rounded to the sample stub using graphite paint, and the specimens were observed under the SEM (S-3000H, Hitachi, Japan) at constant magnifications.

### Assessment of the Leaf Damage

Leaf damage due to herbivory was measured in all treatments by calculating the leaf area of all the leaves on each plant in each treatment. The leaves were put on a Leaf Area Transparent Belt Conveyor (LI-3050C), and it was made sure that the knob was tightened in a way so that the belt moves freely through the scanner head, after the scanner was fixed in the conveyor belt. Leaves were placed on the supporting platform so that they pass through the scanner head and the reading was noted from the display panel. In this way, the leaves from different Si and herbivore treatments were measured.

### Statistical Analysis

Results are reported as means ± SE unless otherwise stated. Data were analyzed using the Student’s *t*-test (*p* ≤ 0.01 and 0.001) and comparison of individual treatment groups was done with one-way ANOVA, and the multiple comparisons where each experimental mean was compared with the control mean were analyzed by Tukey’s *post hoc* test after normality test by the Shapiro-Wilks method. Data showing deviation from a normal distribution were arcsine root transformed before statistical analysis. All the statistical analyses were carried out with SPSS 20.

## Results

### Si Analysis

**Figure 1:**
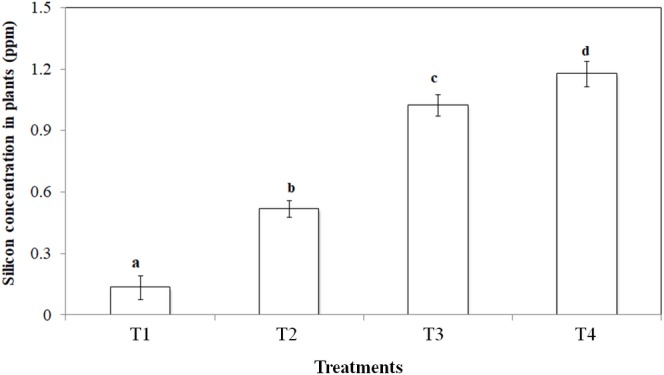
Si uptake potential of *Bromus catharticus* in different Si treatments (T1: No Si; T2: 500 mg/L Si; T3: 1,000 mg/L Si; T4: 2,000 mg/L Si). Values presented are means (*n* = 25). Error bars represent SE. Different letters on top of bars indicate significant differences (*p* < 0.005) between treatments.

Silicon addition to peat increased leaf silicon concentrations significantly (*p* < 0.001). In control treatments, the silicon concentration was 0.1 ppm which increased ca. 12 fold in the highest silicon treatment (Figure 1).

### Phytolith Morphotypes and Epidermal Patterns

**Figure 2:**
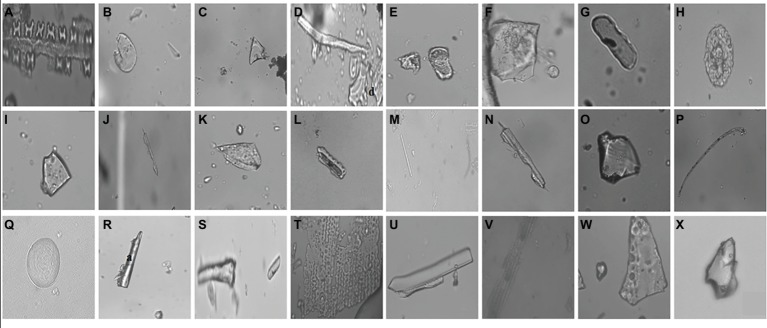
Different types of phytoliths present in *Bromus catharticus*, **(A)** cross shaped, **(B)** orbuscular, **(C)** pyramidal, **(D)** microhair, **(E)** oblong, **(F)** trapezoid, **(G)** oblong elongated, **(H)** globular, **(I)** trapezoid, **(J)** elongated irregular, **(K)** scutiform, **(L)** trapeziform sinuate, **(M)** smooth elongated, **(N)** elongated irregular, **(O)** blocky irregular, **(P)** long hair shaped, **(Q)** orbuscular, **(R)** acicular, **(S)** horn like, **(T)** undulated, **(U**,**V)** rectangular, **(W**,**X)** pyramidal.

**Figure 3:**
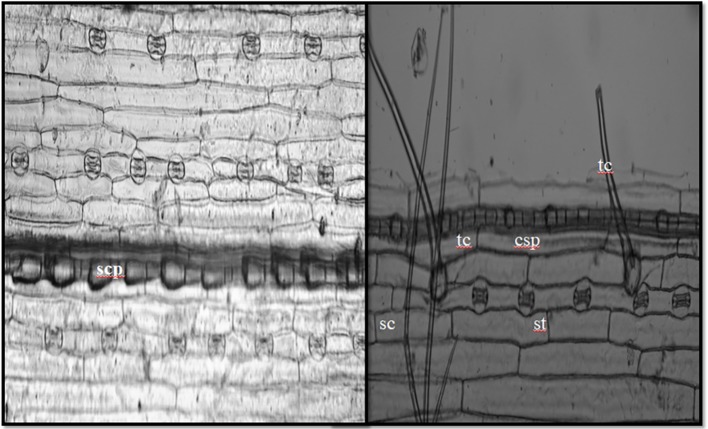
*In situ* location of phytoliths in epidermis of adaxial surface (tc, trichome; sc, short cell phytoliths; st, stomata; sc, silica cells; lc, long cells; scp, short cell phytoliths; cs, cross-shaped phytoliths).

**Figure 4:**
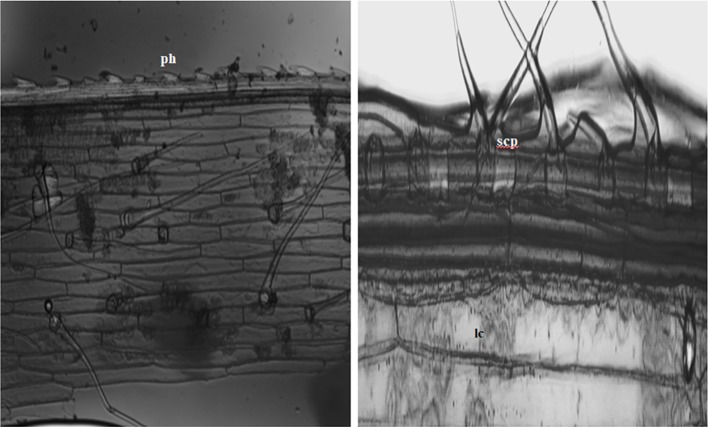
Abaxial surface showing *in situ* silica particles (ph, prickle shaped; lc, long cell; scp, short cell phytoliths) lining the margins of epidermis.

In the current study, we identified a total of 21 phytolith morphotypes in the leaves of *B. catharticus* that were classified into 6 broad groups namely, short cross shaped, epidermal elements, long hairs cells, blocky types, globular, and bulliform cells (Figure 2), that usually originate in the epidermis and endodermis (Twiss et al., 1969; Lu and Liu, 2003). It is pertinent to mention that Si addition did not result in changing the morphology of phytoliths although the frequency of the phytoliths was insignificantly changed. In *B. catharticus,* both the surfaces of the epidermis, i.e., adaxial and abaxial are divided into costal and intercostal zones which differ from each other in cell composition as well as silica deposition. In *B. catharticus,* on the abaxial side, the costal zone is composed of 1–3 layers of cells, and the intercostal zone consists of 4–8 layers of cells (Figure 3). A single layer of cross-shaped silica cells was present in the costal region on the adaxial surface. A smaller number of short cells were present in the intercostal region while the long cells were abundant in both the costal and intercostal regions. On the abaxial side, the costal zone contains 5–6 layers of cells and the intercostal zone consists of 14–18 layers of cells (Figure 4). In the intercostal region, a few cells were shorter than usual long cells. Silicified prickle hairs were present on the leaf border in large numbers along the margin of the leaf.

### Effect of Si Concentration and Grasshopper Herbivory on Rescuegrass Leaf Area

**Figure 5:**
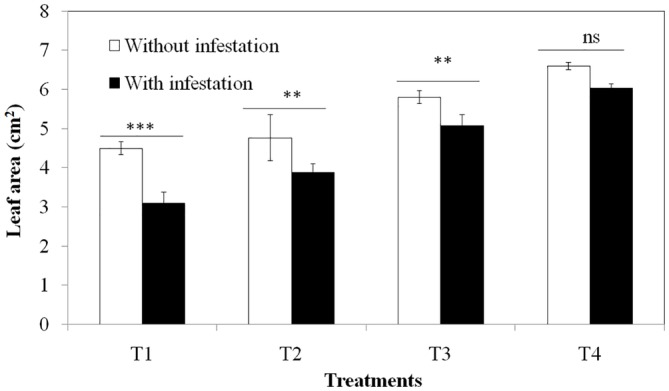
Effect of different Si concentrations (T1: No Si; T2: 500 mg/L Si; T3: 1,000 mg/L Si; T4: 2,000 mg/L Si) on leaf area damage of *Bromus catharticus* due to herbivory. Values presented are means (*n* = 25). Mean values with two or three asterisks are significantly different as determined by the Student’s *t*-test (*p* ≤ 0.01 and 0.001, respectively). Error bars represent SE.

Soil silicon addition had a positive effect on the overall leaf area; however, the consumption of *B. catharticus* by *O. grandis* was reduced in high Si treatments. Higher Si concentrations caused an approximately two fold decrease in herbivory, and the leaf area consumption was similar in the infested and uninfested treatments at higher Si treatments (Figure 5). Hence *B. catharticus* seemed to deter herbivore feeding in high silicon diets by making leaves less palatable for the herbivore to digest.

### Effect of Si Amendments on *O. grandis* Mandible Wear

**Figure 6:**
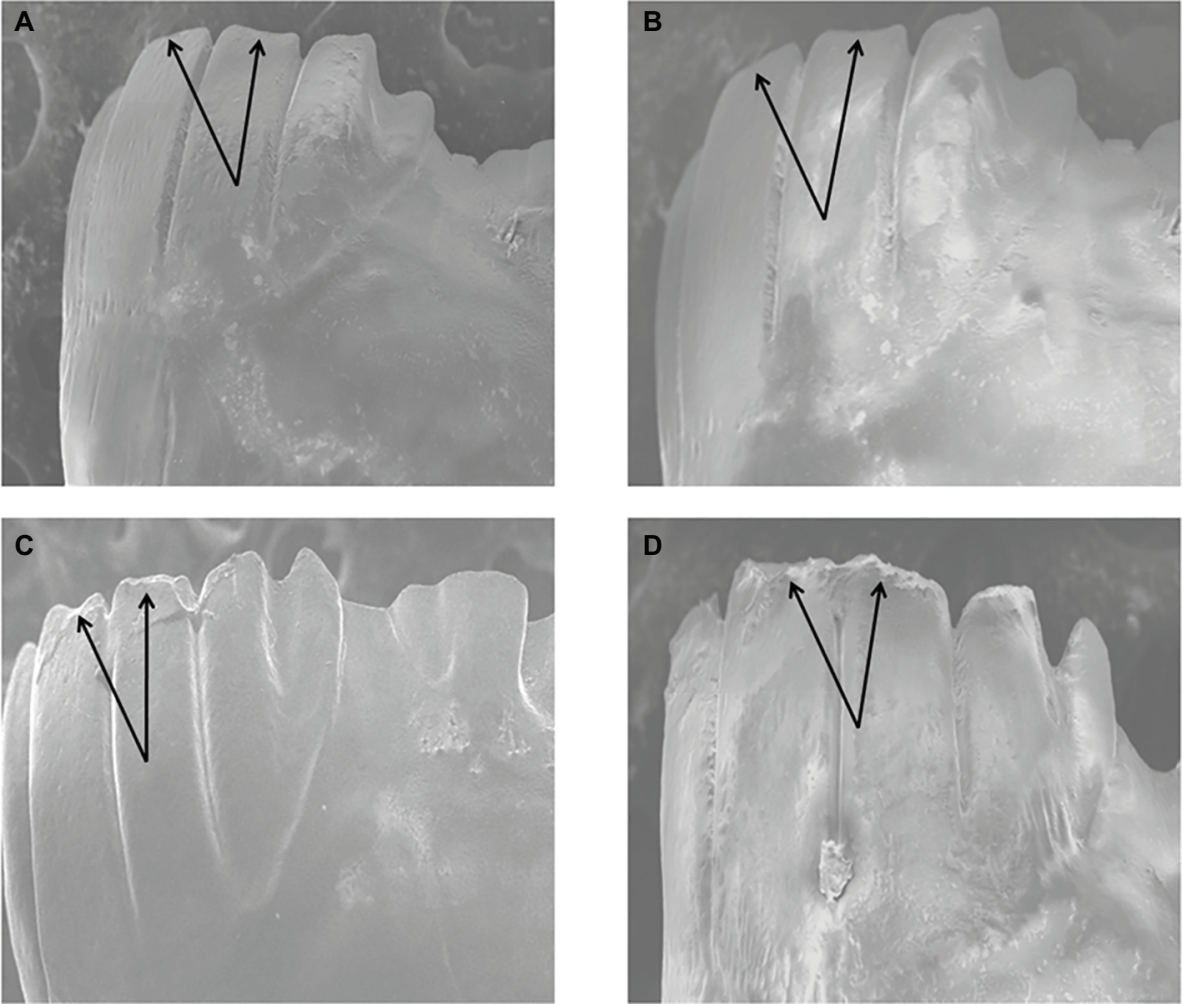
SEM micrographs showing dental wear of *Oxya grandis* mandibles fed on: high Si concentrations **(D),** against control **(A),** and low **(B)** to moderate **(C)** Si concentrations.

The herbivore feeding on a high Si diet showed deformation of the incisors (which is otherwise the strongest part of the mandibles). The results are evident in SEM micrographs (Figure 6).

## Discussion

### Effect of Silicon Concentration and Grasshopper Herbivory on Leaf Area of Rescuegrass

Rescuegrass accumulated a significant amount of Si in Si-rich environments. Based on Si accumulation potential, plants have been categorized into Si accumulators, intermediate type, and excluder species (Jones and Handreck, 1967; Takahashi et al., 1990), and three modes of Si accumulation in plants (active, passive, and rejective) have been proposed for these corresponding types (Takahashi et al., 1990). The resultant Si deposition has been ascribed to high-level phylogenetic position (Hodson et al., 2005). However, the present study clearly demonstrates that Si accumulation also depends on Si availability; although, the source of Si acquired by the plants depends upon the type of minerals absorbed through various processes (Sposito, 2008; Hiradate, 2012). Therefore, the estimation of the Si-supplying power of soils is mainly determined by the available silicon present in the soil (Kyuma, 2004; Sauer et al., 2006; Matsumori and Gunjikake, 2013), and as such the actual potential of accumulation is seldom realized.

### Phytolith Types and Epidermal Pattern

The occurrence of specific phytolith-forming cells in leaves of *B. catharticus* indicates that they are specialized for the defense against insect herbivory. Although Si addition did not change phytolith morphology in the present study, the phytolith frequency (particularly short cell phytoliths) was slightly higher in higher Si treatments. Silicon reduces insect herbivory as it increases epidermal hardness and abrasiveness of the leaf and that provides resistances to the plant and reduces digestibility for the herbivore. The hardness of the plant parts on which herbivore are fed reportedly caused mandibular wear in various beetles (Raupp, 1985; King et al., 1998), bees (Kokko et al., 1993; Schaber et al., 1993), caterpillars (Korth et al., 2006), a locust (Zouhourian-Saghiri et al., 1983), a weevil (Barnes and Giliomee, 1992), and even in the stylet of a true bug (Roitberg et al., 2005). The short cell phytoliths present in the leaf epidermis may discourage both large and small herbivores by making plant tissues less palatable and/or digestible (Hunt et al., 2008; Reynolds et al., 2009) and by wearing down insect mandibles and teeth in mammals (Massey and Hartley, 2009; Müller et al., 2014).

### Herbivore Deterrence

The concentrations of Si in the leaves of *B. catharticus* affected the feeding potential of *O. grandis*. This increased resistance to herbivory has been ascribed to increased abrasiveness and hardness of plant tissues (especially epidermal) due to deposition of silica, mostly in the form of opaline phytoliths (Kaufman et al., 1985; Salim and Saxena, 1992; Ma et al., 2001; Massey et al., 2006; Massey and Hartley, 2009), which might affect the grasshopper directly or indirectly. Si-mediated herbivore resistance acts by hindering the establishment of the insect and defense against plant penetration, which reduces the palatability and feeding efficiency. However, the demonstration of Si-laden plants acting as a mechanical deterrence due to opaline phytoliths is difficult to achieve, and there is significant scope in this research area.

### Mandible Wear

The results of the current study reveal that the long, chisel-edged incisor cusps suffered microwear once the insect fed on plants grown under high silicon treatments. In agreement with previous studies (Sasamoto, 1958; Djamin and Pathak, 1967; Hanifa et al., 1974; Dravé and Lauge, 1978; Zouhourian-Saghiri et al., 1983; Ramachandran and Khan, 1991; Goussain et al., 2002), which reported that insect herbivores feeding on elevated silicon diets suffered greater mandibular wear, the present study showed some microwear in the incisors of the herbivores fed on very high Si treatments, although no mandible wear was witnessed at lower Si concentrations. Studies of dental wear require a minimum of several days of mastication to report measurable mandibular wear, though it mostly depends on the abrasiveness of the foods ingested (Teaford and Glander, 1991, 1996; Gügel et al., 2001). The difference in dental wear could be attributed to higher phytolith content in plants that received higher Si concentrations, as the major mechanical properties controlling abrasiveness of particles are hardness, particle size, and geometry (Williams, 2005). As dental wear markers are often the only proxy system bridging extant biomes and the fossil record, this opens a new research area in the phytolith studies.

## Author Contributions

IR and ZR designed the experiment. SM, IR, RA, and IS carried out the experimental work. BH helped in insect identification and mandible wear study. IR and SM wrote the initial draft of the experiment. SM, IR, BH, and ZR checked the final manuscript.

### Conflict of Interest Statement

The authors declare that the research was conducted in the absence of any commercial or financial relationships that could be construed as a potential conflict of interest.
